# Chemically induced Fermi level pinning effects of high-k dielectrics on graphene

**DOI:** 10.1038/s41598-018-21055-z

**Published:** 2018-02-14

**Authors:** So-Young Kim, Yun Ji Kim, Ukjin Jung, Byoung Hun Lee

**Affiliations:** Center for Emerging Electronic Devices and Systems, School of Materials Science and Engineering, Gwangju Institute of Science and Technology, 123 Cheomdangwagiro, Buk-gu, Gwangju 61005 Republic of Korea

## Abstract

High-k materials such as Al_2_O_3_ and HfO_2_ are widely used as gate dielectrics in graphene devices. However, the effective work function values of metal gate in graphene FET are significantly deviated from their vacuum work function, which is similar to the Fermi level pinning effect observed in silicon MOSFETs with high-k dielectric. The degree of deviation represented by a pinning factor was much worse with HfO_2_ (pinning factor (*S*) = 0.19) than with Al_2_O_3_ (*S* = 0.69). We propose that the significant pinning-like behaviors induced by HfO_2_ are correlated with the oxygen exchange reactions occurred at the interface of graphene and HfO_2_.

## Introduction

Graphene has received considerable attention because of its extraordinary electrical and physical properties, such as its high carrier mobility, excellent thermal stability, and top-down processing compatibility^1–3^. Especially, graphene field-effect transistors (GFETs) have been extensively studied as a promising alternative for post-silicon metal–oxide–semiconductor field-effect transistors (MOSFETs)^4–6^. Recently, graphene barristors and related variations have achieved very high on/off ratios of up to 10^7^ ^7,8^, even without any bandgap. Such progress is motivating further research on gate stacks that are compatible with graphene. Since graphene barristors rely on the sliding of the graphene Fermi level (*E*_F_) at the interface with the semiconductor, it is desirable to minimize the Fermi level pinning in the metal gate/high-k dielectric stack on a graphene channel.

So far, the physical scaling of gate dielectric has been the primary focus of study in the gate stack research for graphene because it is difficult to form a thin gate dielectric on graphene using atomic layer deposition (ALD) process because graphene does not have enough dangling bonds to initiate the chemical reactions for ALD. Thus, various processes have been explored, including surface modification by *in situ* ozone treatments, functionalization of the graphene, or addition of a metal seed layer on the graphene to aid nucleation^9–11^. While the physical scaling of the gate dielectric on graphene has substantially progressed owing to these efforts, the electrical performance—such as quality and reliability—of the gate dielectric has not been thoroughly investigated. Alternatively, hBN has been extensively studied as a thin gate dielectric material for graphene^12–15^. The possibility of direct growth on a graphene due to the small lattice mismatch is a strong merit, but high leakage current with a dielectric constant lower than SiO_2_ (~3–4) indicates that the process is less matured for gate dielectric applications.

On the other hand, the metal gate might be the least studied component in GFET structure. In silicon MOSFETs, the redistribution of oxygen vacancies at the metal gate/high-k dielectric interface causes a work function roll-off phenomenon; this occurs *via* interface dipole formation and Fermi level pinning^16–18^. Furthermore, the redistribution of the oxygen into the substrate strongly influences the effective work function of the metal gate. Thus, gate stacks on GaAs substrates are less influenced by Fermi level pinning than those on silicon, because the oxygen diffusivity in GaAs is lower than that in silicon^19,20^. These findings were critically important for the development of nanoscale silicon MOSFETs, because controlling the effective work function of the metal gate is crucial in controlling the threshold voltage of MOSFETs. Likewise, a proper understanding of the effective work function of the metal electrodes in GFETs will be very important to properly control the Dirac voltage (*V*_Dirac_) of the graphene layers used in various graphene-based devices^21^.

In this work, the interactions between the gate metal, gate dielectric, and graphene in the graphene FETs have been investigated using two different dielectrics, HfO_2_ and Al_2_O_3_, and various metal gates covering a wide range of vacuum work functions from 4.28 to 5.65 eV. We found that the effective work function values of metal gates on the high-k dielectric and graphene stack are significantly deviated from the vacuum work function, which is similar to the Fermi level pinning effect of a metal gate/high-k dielectric stack in silicon MOSFETs. Fermi level pinning factors representing the degree of deviation were extracted from the slope of Dirac voltage and vacuum work function of top gate metal. The pinning factors were ~0.1914 for HfO_2_ and ~0.6912 for Al_2_O_3_, indicating that the Al_2_O_3_ gate dielectric is more suitable for controlling the Dirac voltage of GFETs. Finally, the origin of the Fermi level pinning effect in GFETs was studied using a discharge current analysis (DCA) method.

## Experimental

### Device fabrication

To compare the influences of different metal gates and high-k dielectrics on multiple GFETs, the baseline GFETs used in this work should have relatively small scattering in the device characteristics. Especially, the device should show symmetric I-V characteristics to accurately assess the impact of Fermi level pinning using the distribution of Dirac points. We applied various addition annealing and passivation processes to achieve symmetric I-V characteristics with minimal hysteresis and residual charges as described below^22^.

The 90 nm SiO_2_/highly p-doped silicon substrate (resistance < 0.005 Ω) was sonicated sequentially in acetone, methanol, and deionized (DI) water. The substrate was then cleaned using the SC1 process (NH_4_OH, H_2_O_2_, and H_2_O in a ratio of 1:1:5 at 80 °C for 10 min). Monolayer graphene was grown on a Cu foil (≥99.8%) using thermal chemical vapor deposition (CVD), and then transferred to the SiO_2_ substrate. First, the graphene was coated with poly(methyl methacrylate) (PMMA) at 500 rpm for 5 s and 3000 rpm for 50 s, and then baked at 80 °C for 5 min. Then, the Cu foil was etched at 50 °C using a Cu etchant. The PMMA/graphene film was cleaned with diluted HCl (HCl and H_2_O in a ratio of 5:13) then rinsed with DI water. A final DI water rinse was performed for 5 min, and then the PMMA/graphene film was transferred to the substrate and dried overnight.

**Figure 1:**
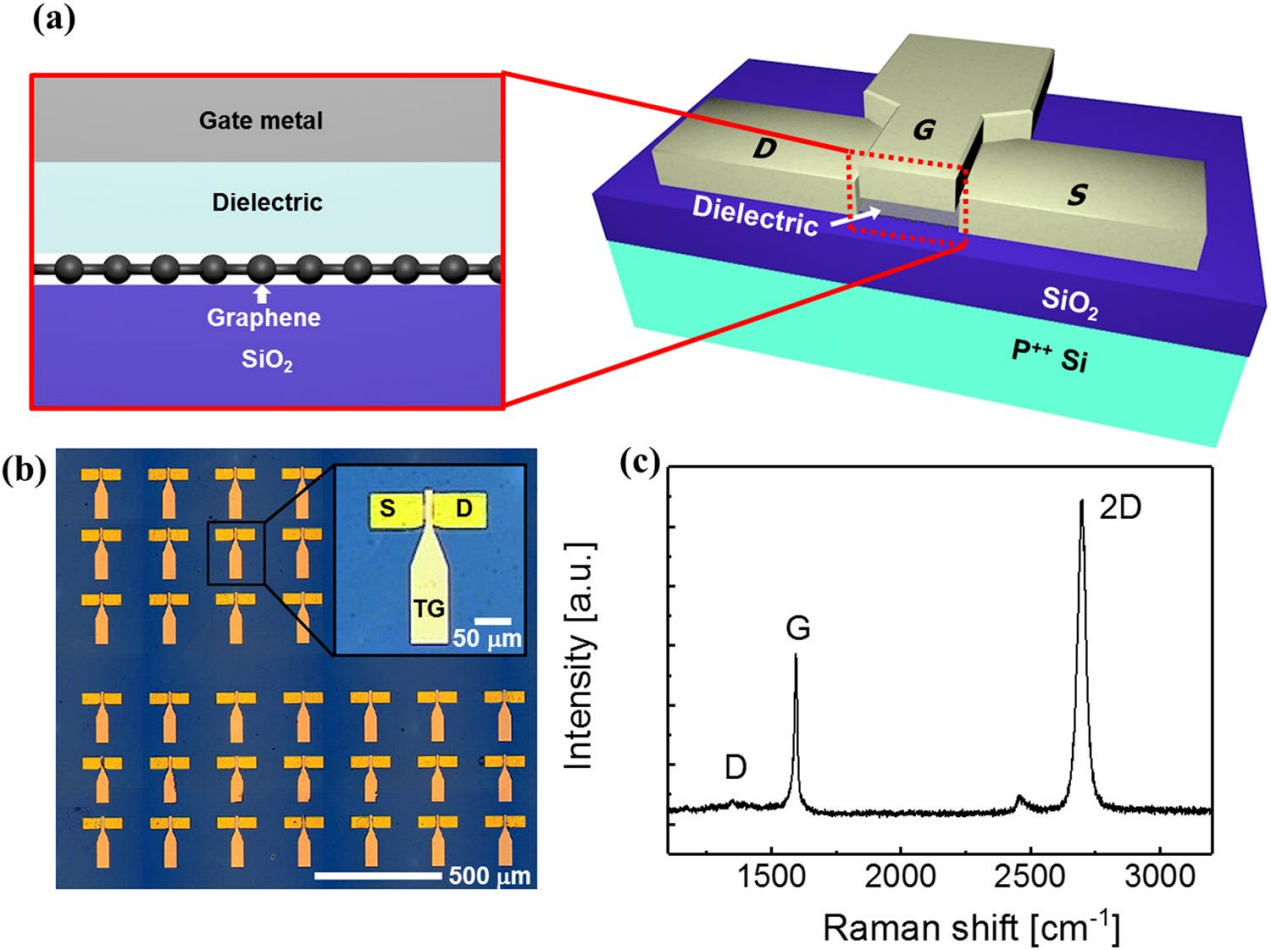
Structure and electrical characteristics of top gated GFET; (**a**) Top gated GFETs were fabricated with different high-k dielectrics such as HfO_2_ and Al_2_O_3_. (**b**) Optical image of fabricated top gated GFETs in a sample and inset figure emphasize the structure of single device. (**c**) Raman spectrum of CVD graphene and it shows transferred graphene is monolayer with low defects.

To remove the PMMA layer, the samples were baked at 150 °C for 10 min and then submerged in acetone heated to 85 °C for 3 hr. Finally, thermal annealing was performed at 300 °C for 1 hr in a vacuum (~10^−6^ Torr). The Raman spectrum of the transferred graphene fits well with a Lorentzian function. As shown in Fig. 1(c), the D/G integrated peak area ratio was ~0.15 and the 2 D/G integrated peak area ratio was 4.11, indicating the presence of high-quality monolayer graphene.

For the device fabrication, a 20 nm-thick Au layer was deposited on the graphene using an electron beam (e-beam) evaporator, followed by the patterning of the channel and contact regions by photolithography and an Au etchant. Then, the graphene was etched by oxygen plasma according to the Au hard mask, which was performed at 50 W in a 200 mTorr environment for 2 min. The final graphene channel width and length was 30 µm and 9 µm, respectively. Then, the source/drain electrodes (100 nm Au) were formed by photolithography and a metal wet etch process.

Al_2_O_3_ or HfO_2_ was deposited on the graphene channel as a top-gate dielectric using ALD. For HfO_2_, a Hf seed layer (2 nm) was deposited using the e-beam evaporator because of the nonuniform nucleation that arises without the seed layer^11^. Before the ALD process, the Hf seed layer was oxidized using 10 s H_2_O/30 s N_2_ purge steps (10 cycles) at 100 °C. Then, HfO_2_ was deposited over 400 ALD cycles using tetrakis(ethylmethylamino)hafnium (TEMAHf, 0.2 s)/N_2_ purge (10 s)/H_2_O (0.2 s)/N_2_ purge (10 s) steps at 250 °C. In the case of Al_2_O_3_, the trimethylaluminum (TMA, 0.2 s)/N_2_ purge (20 s)/H_2_O (0.2 s)/N_2_ purge (20 s) cycle was repeated 300 times at 130 °C. Both dielectrics were densified at 300 °C for 1 hr under vacuum (~10^−6^ Torr). The thicknesses of the Al_2_O_3_ and HfO_2_ layers measured with a spectroscopic ellipsometer were both ~30 nm. Finally, four different top-gate metals—Pt, Au, Ti, and Al—were deposited and pattered using a lift-off process. The top-gate metals were chosen so their work functions covered the range from 4.28 to 5.65 eV. Figure 1(a) schematically illustrates the completed top-gated GFET device structure and Fig. 1(b) shows the optical image for top gated GFET. The completed devices were annealed under vacuum (~10^−6^ Torr) at 300 °C for 1 hr before the electrical tests were performed.

### Electrical characterization

The device characteristics were measured using a Keithley 4200 Parameter Analyzer with a pulsed *I–V* measurement unit (Keithley 4225-RPM)^23–26^. A pulsed *I*–*V* measurement method was used to minimize the influence of slow charge trapping effects. The pulse width and rise/fall time was 1 ms and 100 µs, respectively, which were the optimized test conditions for the top-gated GFETs used in this work. The gate voltage was increased in one direction starting from near the Dirac voltage to minimize hysteresis. Since the dielectric constant of Al_2_O_3_ is 6 and that of HfO_2_ is 20.6, the gate voltages were biased from −10 V to 10 V for the Al_2_O_3_ device and from −2.9 V to 2.9 V for the HfO_2_ device to generate matching maximum electric fields of around ±5.1 MV/cm. The mobility, hysteresis-induced charge trap density, and Dirac voltage was extracted from the electron-doped branch of the pulsed *I*_d_–*V*_g_ curve. The charge trap density of the top-gated GFETs at the Fermi level of graphene was extracted using the DCA method^25^. For the DCA measurements, square pulses were applied to the top-gate, ranging from 10 kHz to 1 MHz for Al_2_O_3_ and from 10 kHz to 500 kHz for HfO_2_. The frequency ranges were adjusted considering the influence of the different time constants of the charge traps in each dielectric. The capacitance–voltage curves of the top-gated GFETs were also measured using an impedance analyzer (Agilent 4294 A) to extract the *E*_F_ value of graphene using the quantum capacitance. A detailed description of the DCA setup and analysis is given in Ref.^25^.

## Results and Discussion

**Figure 2:**
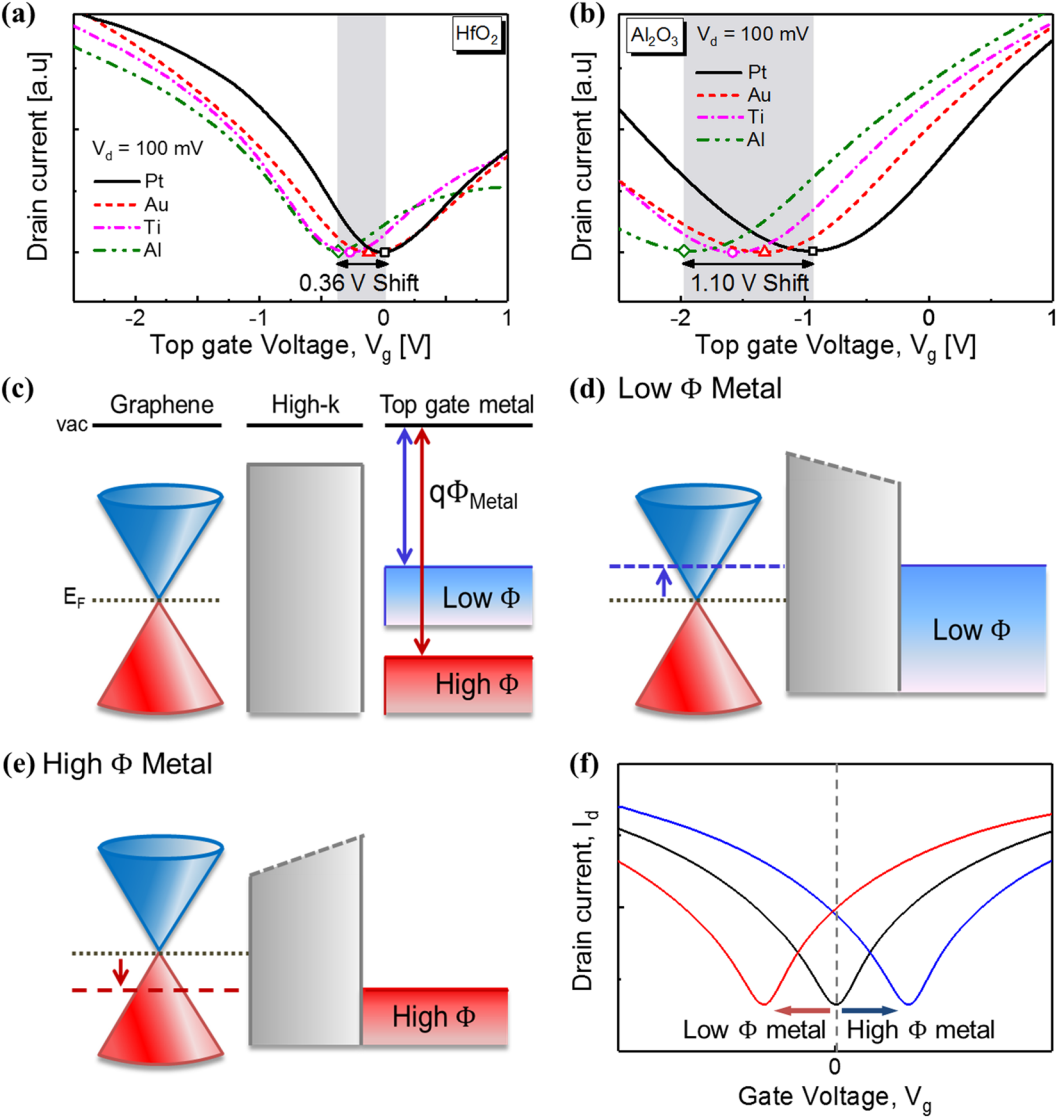
Band diagram and electrical characteristics of top gated GFET with various top metals; (**a**,**b**) Pulsed *I*_d_ – *V*_g_ characteristics of top gate GFETs, which were fabricated with different top dielectrics such as (**a**) HfO_2_ and (**b**) Al_2_O_3_, when V_d_ = 100 mV. (**c**) Metal/Oxide/Graphene band diagram of top gated GFET with various top metals, which have low or high work function. When gate voltage is biased, graphene should electrically doped by difference of work function between graphene and metal. (**d**) When the metal work function is smaller than graphene, graphene should be electron doped. (**e**) On the contrary, graphene should be hole doped with a high work function such as Au and Pt. (**f**) When graphene is electrically doped, V_Dirac_ in *I*_d_ – *V*_g_ characteristics should be shifted.

The representative pulsed drain current–gate voltage (*I*_d_ – *V*_g_) curves of the top-gated GFETs having different top-gate metals are shown in Fig. 2(a). The minimum current at the Dirac point varied slightly from sample to sample. Assuming that the total effective residual charge densities and mobility of the top-gated GFETs were not directly influenced by the work function of gate metal (Supplementary Fig. S1), the drain currents were divided by the minimum off current to show the lateral shifts in Dirac voltages clearly, as shown in Fig. 2(b). The Dirac voltage gradually shifted to more positive values as the work function of the gate metal increased. Interestingly, the extent of the shift strongly depended on the gate dielectric. The shift was only 0.22 V for HfO_2_, which is much smaller than that expected based on the range of vacuum work function values. In contrast, the range of Al_2_O_3_ Dirac voltages was 1.10 V. This behavior is somewhat similar to the Fermi level pinning effects observed in silicon MOSFETs which limits the swing range of the threshold voltage so that it is much smaller than the range of the vacuum work function of metal gates. However, since the Fermi level pinning in silicon MOSFETs is related to trap states at the dielectric/silicon interface, whether we can attribute this observation to Fermi level pinning remains uncertain.

The schematic band diagrams of the metal/oxide/graphene structure shown in Fig. 2(c–f) illustrate how the *I*_d_ – *V*_g_ curves are shifted by the changes in the metal gate work function. For this discussion, we assume the intrinsic work function of graphene to be 4.6 eV^27^. When the work function of the top metal is smaller than 4.6 eV, graphene is electron doped, as shown in Fig. 2(d). As a result, the Dirac points of the *I*_d_ – *V*_g_ curves are negatively shifted, as shown in Fig. 2(e). Conversely, when the work function of the top metal is higher than 4.6 eV, graphene is hole doped, as shown in Fig. 2(f), and the Dirac points of the *I*_d_ – *V*_g_ curves are positively shifted.

In silicon MOSFETs, the interface states at the silicon substrate surface limits the range of the Fermi level shift. When a high-k dielectric is used, the dipoles formed at the interface between the high-k dielectric and interfacial oxide shift the effective work function of metal gate. In addition, when a metal gate is used, the oxygen exchange between the metal, high-k dielectric, and silicon substrate shifts the effective work function of the metal by changing the oxygen stoichiometry at the multiple interfaces within the gate stack. All of these factors that affect the final manifestation of the effective work function are often summarized as a pinning effect. The term “pinning” is originally from the Bardeen limit condition of metal/semiconductor Schottky contact where the location of the work function of metal is pinned to the midgap level of silicon energy band due to the charge exchange with interface states of silicon. Since the origin of the work function modulation in our device is not related to the interface states, “pinning” is not an exactly correct technical term, but nowadays, it is often used as a term representing the systematic deviation of effective work function from the vacuum work function. This situation can be described using the equation (1).

1 $${{\rm{\Phi}}_{{\rm{M}},{\rm{eff}}}={{\rm{\Phi}}_{{\rm{CND}},{\rm{d}}}+S({{\rm{\Phi}}_{{\rm{M}},{\rm{vac}}}-{{\rm{\Phi}}_{{\rm{CND}},{\rm{d}}})}}}}$$

where Ф_M,eff_ is the effective work function of the metal gate, Ф_M,vac_ is the work function of the metal gate in a vacuum, Ф_CND,d_ is the charge neutrality level of the dielectric. When S = 1, the effective work function is equal to the vacuum work function^28^. On the other hand, as the S decreases, the effective work function approaches to the dielectric specific work function value regardless of the initial vacuum work function.

There are several well-known mechanisms that modulate the effective work function of metal in metal/oxide/silicon structure such as the excessive interface states, the dipoles at the interface of metal/high-k dielectric due to the oxygen redistribution, the dipoles at the interface of high-k dielectric/silicon due to the charge redistribution^29,30^. However, these mechanisms may not be directly applicable for an ideal metal/oxide/graphene structure, because high-quality epitaxial graphene does not have interface states arising from dangling bonds and interfacial oxides. Furthermore, oxygen penetration through the graphene should be minimal. Thus, oxygen redistribution can only occur at the metal/high-k dielectric interface. As a result, the work function shift in a GFET should be less significant, as in the case of the high-k dielectric on GaAs.

**Figure 3:**
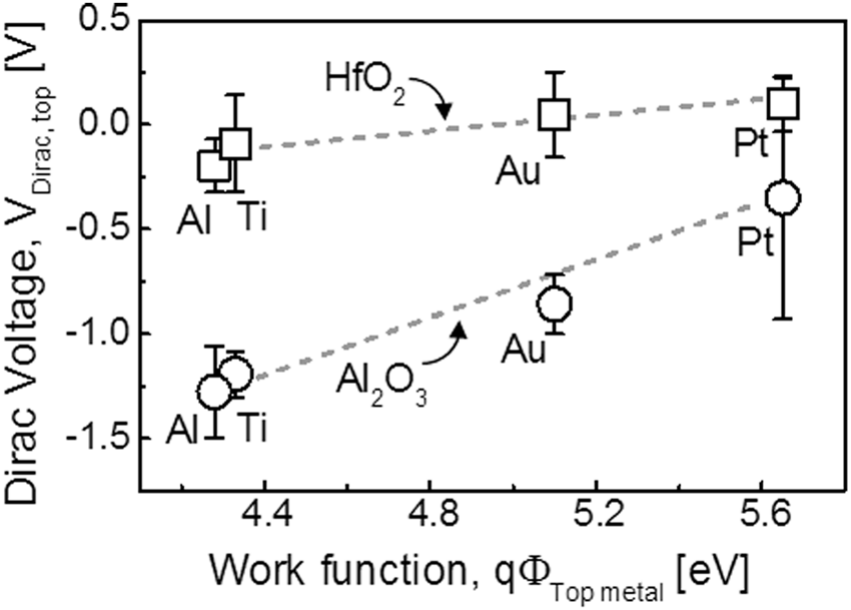
Dirac voltages of top gated GFET depending on top metal work function; Dirac voltages of top gate GFETs, which were integrated with Al_2_O_3_ (circle) or HfO_2_ (square), are shifted depending on the top metal work function. Pinning factor (S) was extracted from the slope of linear fitting (gray dot) as 0.1914 for HfO_2_ and 0.6912 for Al_2_O_3_.

Despite of these expectations, Fermi level pinning–like behaviors were observed when the Dirac voltages of the GFETs were correlated with the vacuum work function of the gate metal, as shown in Fig. 3. The range of the Dirac voltage values was approximately 1.09 V for Al_2_O_3_, but only 0.297 V for HfO_2_. Assuming the effect of charge trapping was minimized by the pulsed *I–V* measurements, the Dirac voltage shift extracted from the *I*_d_–*V*_g_ curves can be primarily attributed to the change in the effective work function. Hence, the pinning factor *S* can be extracted using Equation (2).

2 $${{\rm{\Delta }}V}_{{\rm{Dirac}}} \sim {{\rm{\Delta }}{\rm{\Phi}}_{{\rm{M}},{\rm{eff}}}}={S}\times {{\rm{\Delta }}{\rm{\Phi}}_{{\rm{M}},{\rm{vac}}}}$$

The values of *S* obtained from the slopes of the Dirac voltage versus vacuum work function curves were 0.69 for Al_2_O_3_ and 0.19 for HfO_2_. The restricted effective work function range, *i*.*e*., Dirac voltage shift, is a typical characteristic of the Fermi level pinning effect^31^. Nevertheless, the presence of a Fermi level pinning–like phenomenon at the metal/high-k dielectric stack on graphene is very intriguing because it does not follow the theoretically predicted behavior, as mentioned above. The typical pinning factor for HfO_2_ in a silicon MOSFET is 0.52, while that for Al_2_O_3_ is 0.69. Interestingly, the pinning factors for Al_2_O_3_ in both silicon FETs and GFETs are similar. In contrast, the pinning factor for HfO_2_ was significantly smaller, *i*.*e*., the Fermi level pinning like effect is more severely manifested with HfO_2_.

To investigate this interesting phenomenon, the differences in the graphene and high-k dielectric interfacial reactions were investigated using the DCA method^24–26^. DCA can extract the number of charge trapping–like events that occur in or near the graphene by monitoring the frequency dependence of discharging current from the graphene channel which was charged by the square pulses applied to the gate. More specifically, two types of charge traps—tunneling and reduction–oxidation (redox) reactions—can be measured separately according to the response time; redox components primarily appear at higher frequencies^23^. Moreover, the quantities of these two types of charge generation mechanism can be extracted using the DCA method.

**Figure 4:**
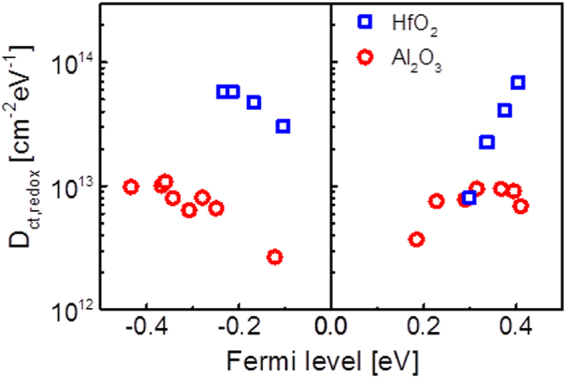
Charge trap density of top gated GFETs with two different high-k dielectrics; Density of charge trap (D_ct_) through redox component depends on Fermi level of graphene for two kinds of top gated GFETs, which fabricated with HfO_2_ (blue square) and Al_2_O_3_ (red circle). Top gated GFET with HfO_2_ has 10 times higher redox charge trap densities than Al_2_O_3_ device.

Figure 4 shows the density of the redox-related charge traps as a function of the Fermi level. GFETs with Al_2_O_3_ gave a charge density that was ten times lower than that of HfO_2_. This result indicates that there are significantly more oxygen-related charge generation events at the HfO_2_/graphene interface. This is a reasonable result considering that oxygen ions diffuse much faster in HfO_2_ than in Al_2_O_3_^32,33^. Oxygen vacancies in dielectrics can create an interfacial dipole layer through electron transfer that affects the Fermi level pinning^34,35^. Hence, the high density of charge traps in the top-gated GFETs containing HfO_2_ corresponds with the small pinning factor extracted from the relationship between the Dirac voltage and the work function of the top-gate metal.

**Figure 5:**
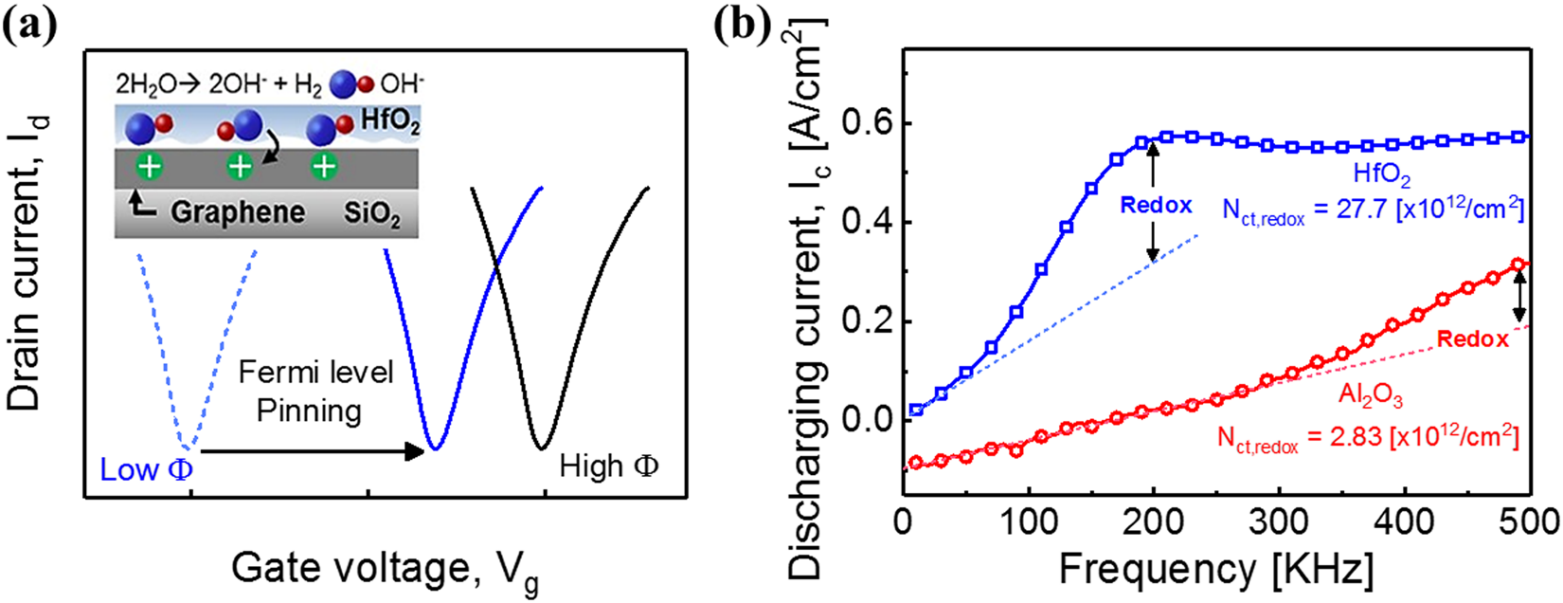
Mechanism of chemically induced Fermi level pinning effects in top gated GFET and results of DCA measurement; (**a**) Illustration of the mechanism about electron doping prohibition at graphene and dielectric interface. Strong OH^−^ charges at the interface inhibits electron doping of graphene. (**b**) *I*_c_ – *f* curve of top gated GFETs using Al_2_O_3_ and HfO_2_. Top gated GFET with HfO_2_ has steeper slope and higher defect density of redox component than the top gated GFET with Al_2_O_3_.

Especially, oxygen accumulated at the bottom interface of HfO_2_ can affect the Fermi level pinning. Figure 2(a) shows that the restriction of the *I–V* curve shift *via* Fermi level pinning is more pronounced at negative biases, *i*.*e*., lower work functions. This result implies that the electron doping of the graphene is weaker than expected for the lower work function metal gates, i.e. there is a mechanism prohibiting the electron doping of the graphene, especially in the case of HfO_2_, as graphically shown in Fig. 5(a). Previously, we have reported that water redox reactions can induce strong OH^−^ charging at the graphene surface and thus prevent electron doping. Figure 5(b) shows that there is indeed a strong redox reaction component in the DCA for HfO_2_, in contrast to Al_2_O_3_, providing corroborating evidence for our model. Thus, we can conclude that the strong Fermi level pinning observed for the GFETs fabricated with the HfO_2_ gate dielectric is due to the inhibition of electron doping, which occurs *via* counter-charge generation at the dielectric/graphene interface due to the interfacial oxygen accumulation. This mechanism is quite different from the original Fermi level pinning mechanism because the chemically induced charges are limiting the movement of Fermi level of graphene rather than the defect states.

## Conclusion

In this research, the dependence of the Fermi level pinning effect on the dielectric was investigated using pulsed current–voltage and DCA methods. From the analysis of our measurements, the pinning factor and charge trap density induced by the redox component (*D*_ct,redox_) in the top-gated GFETs with HfO_2_ were 0.1914 and ~10^14^ cm^−2^ eV^−1^, respectively. These values show that HfO_2_ devices experience a more significant pinning effect than Al_2_O_3_ devices, which have a pinning factor of 0.6912 and *D*_ct,redox_ = ~10^13^ cm^−2^ eV^−1^. These results indicate that the pinning effect is caused by redox reactions at the interface between the high-k dielectric and graphene. These results indicate that the scaling of gate dielectrics using higher-k materials will be more difficult for GFETs and will require stringent control over the interface and its passivation.

## Electronic supplementary material

Figure S1
