# Supplementary material for: Chemically induced Fermi level pinning effects of high-k dielectrics on graphene
Source: Sci Rep. 2018 Feb 14;8:2992. doi: 10.1038/s41598-018-21055-z (PMC5813236; doi:10.1038/s41598-018-21055-z)
Supplement: Supplementary file 1 — Figure S1 [file 41598_2018_21055_MOESM1_ESM.doc]

***Supplementary information***

***Chemically induced Fermi level pinning effects of high-k dielectrics on graphene***

So-Young Kim, Yun Ji Kim, Ukjin Jung, and Byoung Hun Lee*

Center for Emerging Electronic Devices and Systems, School of Materials Science and Engineering, Gwangju Institute of Science and Technology, 123 Cheomdangwagiro, Buk-gu, Gwangju 61005, Republic of Korea

Corresponding author: Byoung Hun Lee (bhl@gist.ac.kr)

**Table of Contents**

**Charge density and mobility of top gated GFETs (Figure S1) S3**

**Charge density and mobility of top gated GFETs**

**
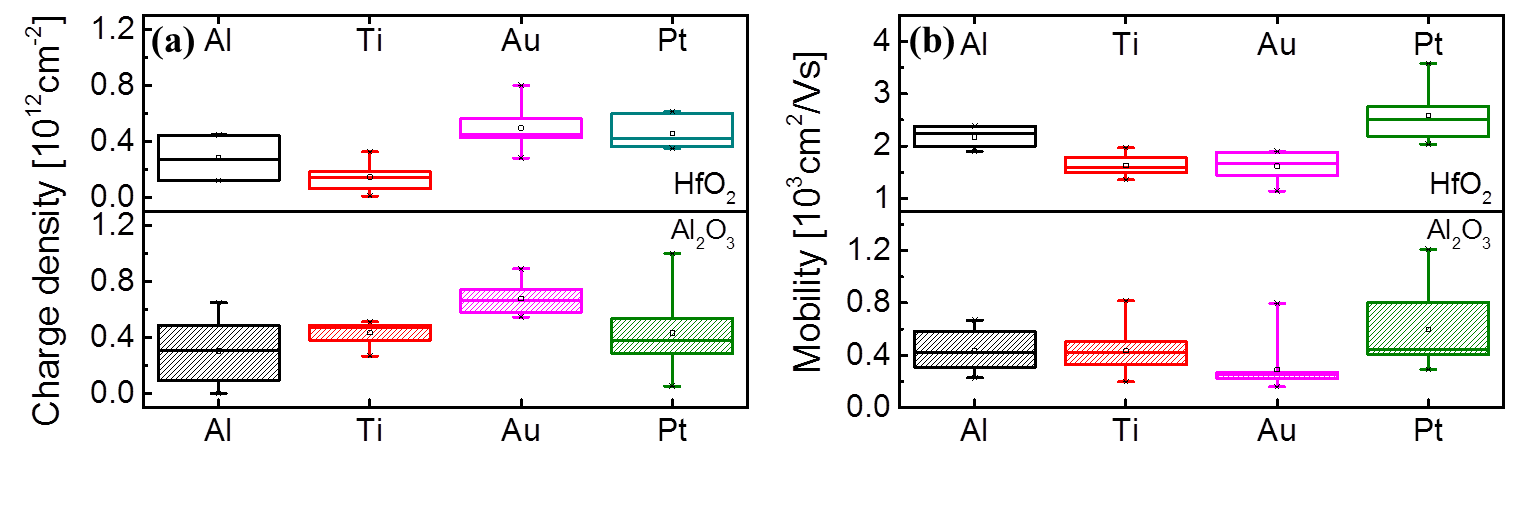
Figure S1 Electrical characteristics of top gated GFETs. a,** Total effective residual charge density (
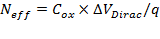
) and **b,** field effect mobility (
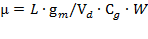
), where W and L are channel width and length, C_g_ is the capacitance per unit area of gate oxide, V_d_ is drain voltage, and g_m_ is the
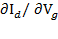
.
